# Mid-infrared optical modulator enabled by photothermal effect

**DOI:** 10.1038/s41377-022-01059-1

**Published:** 2023-01-02

**Authors:** Zhen Wang, Wei Ren

**Affiliations:** Department of Mechanical and Automation Engineering, The Chinese University of Hong Kong, New Territories, Hong Kong SAR, China

**Keywords:** Fibre optics and optical communications, Optical manipulation and tweezers

## Abstract

Photothermal effect in a gas-filled hollow-core fiber may result in agile mid-infrared optical modulators for broadband phase modulation and high extinction ratio intensity modulation.

Photothermal effect refers to the heat generation caused by the absorption of light, which has widespread applications in imaging, spectroscopy and medical therapy. In particular, the use of photothermal effect has become one of the most effective methods for microscopic imaging and spectroscopic analysis. The typical photothermal system involves a pump-probe optical configuration. When the light of an intensity- or frequency-modulated pump laser is absorbed, the subsequent relaxation process disperses the internal energy to cause periodic heating of the surrounding medium. Mid-infrared (MIR) photothermal imaging can exceed the diffraction limit of infrared microscopy and allow label-free three-dimensional chemical imaging of live cells and organisms^1^. By taking advantage of the photothermal effect that combines high-frequency modulation and polarization interference contrast, researchers have demonstrated the far-field optical detection of gold colloids down to diameters of 2.5 nm^2^. Compared to liquid and solid with strong absorption, it is becoming more challenging to analyze highly diluted gas samples. One of the first attempts to use photothermal effect for quantitatively measuring gas species was demonstrated in 1981 by using a 20 W CO_2_ gas laser as the pump source^3^. However, gas-phase photothermal spectroscopy has limited applications due to the requirement of high-power pump lasers and the complex configuration of arranging pump and probe beams.

It is of particular interest to explore new methods of achieving sufficient photothermal effect in the gas-phase medium based on low-power pump light sources such as semiconductor lasers. Fortunately, a breakthrough was made in this area by confining the laser beams and gas molecules in a tiny volume such as a hollow-core fiber (HCF). By taking advantage of a hollow-core photonic bandgap fiber with an 11-μm core diameter, Jin et al. demonstrated ultrasensitive photothermal detection of acetylene (2 ppb) using a near-infrared (NIR) external-cavity diode laser with a pump power of 15.3 mW^4^. This innovation completely relaxes the restriction of using various types of low-power pump lasers for sensitive photothermal spectroscopy. Recently, Wang et al. demonstrated dual-comb photothermal spectroscopy for broadband, sensitive and high-precision molecular spectroscopy by successfully detecting the frequency-comb-induced photothermal modulations in an HCF^5^. Thanks to the recent advance of fiber technology, MIR-HCFs have enabled more sensitive gas-phase photothermal spectroscopy by exploiting the molecular fingerprint spectral region^6,7^.

Is it possible to extend the photothermal effect to other applications such as optical modulators? An optical modulator is a device that is used to manipulate light properties such as the optical phase, amplitude and polarization of a light beam propagating either in free space or in an optical waveguide. This key component is widely used in optical communication, photonic circuits, data encoding, active Q-switched laser generation, and optical metrology and spectroscopy. Depending on whether the absorption coefficient or the refractive index of the material is changed to modulate the light beam, optical modulators can be classified as either absorptive or refractive type^8^. To date, commercial optical modulators are mainly established on waveguide-integrated devices with silicon-on-insulator and silicon-on-lithium-niobate configurations. However, their use is limited in the telecom NIR region due to strong material absorption at longer wavelengths.

Now, writing in Light: Advanced Manufacturing, a sister journal of Light: Science & Applications, Prof. Wei Jin and his team from the Hong Kong Polytechnic University reported an experimental realization of a novel MIR optical modulator enabled by the photothermal effect in an acetylene-filled anti-resonant hollow-core fiber (AR-HCF)^9^. With the assistance of a NIR control beam, the phase of the signal beam is observed to be directly modulated up to 2.2π rad in the MIR (3.35 μm), which extends their previous work on all-fiber phase modulator within C + L bands successfully^10^. And the intensity modulation is verified with a high extinction ratio of 25 dB by employing a Mach-Zehnder interferometer (MZI).

The working principle of the photothermal-based optical modulator is shown in Fig. 1. Optical absorption of the NIR control beam by acetylene molecules changes the refractive index of the gas medium inside the HCF, which modulates the accumulated phase of the MIR signal beam propagating in the same HCF. In the work, a distributed feedback diode laser is used as the control light and its wavelength is tuned to the P(9) line of acetylene at 1530.371 nm. As a proof-of-principle experiment, an interband cascade laser at 3.35 μm is used as the signal beam to characterize the modulation performance. By filling pure acetylene in the HCF at the pressure of 2 bar and modulating the NIR control laser at 3 kHz, experimental results indicate that the phase modulation is over π rad for a control light power of 280 mW. The phase modulation can be increased to 1.34 π rad by using a higher control power of 650 mW. Furthermore, the phase modulation depends on the gas pressure in the HCF. By increasing the pressure to 3.4 bar, the authors obtain a large phase modulation of 2.2 π rad, which is mainly due to the increased gas molecular density inside the HCF. In addition, by placing the phase modulator in one arm of an MZI, an optically controlled MIR intensity modulator can also be realized. Compared to the commercial modulator with a damage threshold of ~1 W/mm^2^, the gas-filled HCF device can increase the threshold by nearly 6 orders of magnitude^11^.

**Fig. 1:**
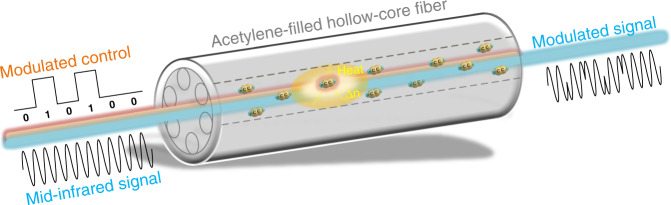
Schematic of mid-infrared optical modulator enabled by photothermal effect in a gas-filled hollow-core fiber

The successful realization of the MIR modulator demonstrates the feasibility of using the gas-filled HCF as a promising platform, which significantly expands the scope of MIR modulators to achieve new photonic functionalities. It should be noted that the modulation frequency of the gas-filled HCF modulator is currently limited to kHz-level, slower than that of crystal or waveguide-based modulators. It is possible to alleviate this issue by using HCFs with different microstructures to enhance the heat conduction process. Attributed to the benefits of HCFs with broadband low-loss transmission range, nearly perfect overlap of the control-signal light fields, and extremely narrow lines of acetylene, the proposed method is also promising for ultra-broadband modulator development from ultraviolet to far-infrared wavelength regions. This will open the door to many applications where the broadband property is critical, such as actively mode-locked lasers, wideband laser frequency stabilization, and other photonics and sensing applications.
